# Quasicrystal metasurface for optical holography and diffraction

**DOI:** 10.1038/s41377-024-01578-z

**Published:** 2024-09-09

**Authors:** Xin Wen, Zixian Hu, Heng Wang, Yu Chen, Qichang Ma, Guixin Li

**Affiliations:** 1 Department of Materials Science and Engineering, Southern University of Science and Technology, Shenzhen, China; 2 Institute for Applied Optics and Precision Engineering, Southern University of Science and Technology, Shenzhen, China

**Keywords:** Sub-wavelength optics, Nanophotonics and plasmonics

## Abstract

Quasicrystal metasurfaces, a kind of two-dimensional artificial optical materials with subwavelength meta-atoms arranged in quasi-periodic tiling schemes, have attracted extensive attentions due to their novel optical properties. In a recent work, a dual-functional quasicrystal metasurface, which can be used to simultaneously generate the diffraction pattern and holographic image, is experimentally demonstrated. The proposed method expands the manipulation dimensions for multi-functional quasicrystal metasurfaces and may have important applications in microscopy, optical information processing, optical encryption, etc.

Holography, is a technique first introduced by Denis Gabor in 1948 to improve the resolution of electron microscope^1^. Later, optical holography was extensively investigated in many areas, including microscopy^2^, three-dimensional display^3^, classical and quantum optical information processing^4,5^. Compared to conventional optical holographic devices such as spatial light modulators^6^ and multilevel diffractive optical elements^7^, metasurface composed of spatially variant meta-atoms represents a novel platform for manipulating the degrees of freedom of light fields^8^. As the feature size and phase steps of the meta-atoms can be easily engineered, metasurfaces usually have higher optical diffraction efficiency than their counterparts. With the rapid development of the design principles of meta-atoms, the efficiency^9^, multiplexing channels^10^ and working bandwidth^11,12^ of the metasurface optical holograms have been greatly improved in the past years. Furthermore, employing the concept of nonlinear geometric phase^13,14^, vectorial holographic images at second harmonic frequencies are demonstrated by using the nonlinear plasmonic metasurfaces^15^.

Inspired by the concept of quasicrystal^16,17^, various photonic quasicrystals^18^ are proposed to control the properties of light transmission^19^, laser action^20^ and harmonic generations^21,22^. Usually, the optical properties of photonic quasicrystals are mainly governed by the long-range order without translational periodicity. On the other side, the phase, polarization and amplitude of light can be locally manipulated by using either plasmonic or dielectric meta-atoms^8^. Therefore, more optical functionalities can be developed by taking the advantages of photonic quasicrystal and metasurfaces.

In a recent work published in *eLight*^23^, Xu et al. propose and demonstrate a Penrose type quasicrystal metasurface which can be used to simultaneously reconstruct holographic images and project far-field diffraction patterns, as illustrated in Fig. 1. The proposed quasicrystal metasurface is composed of silicon meta-atoms which are fabricated on a glass substrate by using electron beam lithography and inductively coupled plasma etching. By judiciously designing the distributions of the geometric phase and propagation phase type meta-atoms by using a self-developed holographic algorithm, the authors realize the dual functionality of optical holography and diffraction. In principle, different tiling schemes^22,24^ and optical properties can be introduced into the design of the quasicrystal metasurfaces. It is anticipated that the proposed strategy may have important applications in the areas of optical information encryption, optical display, optical computing and so on.

**Fig. 1:**
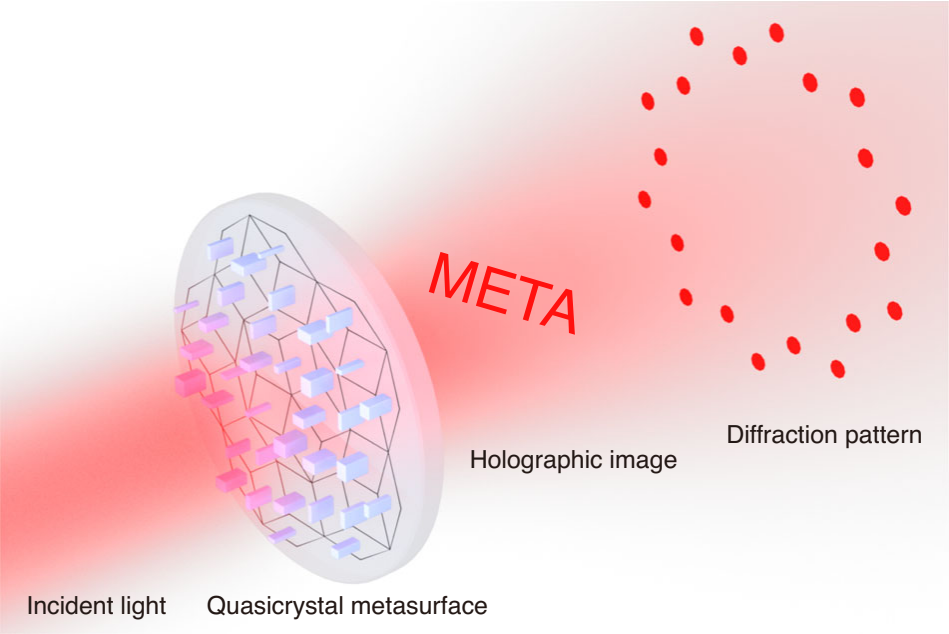
Schematic illustration of the quasicrystal metasurface for optical holography and diffraction. The quasicrystal metasurface consists of silicon meta-atoms arranged based on the Penrose tiling scheme. Under the illumination of the normally incident light, a holographic image at the predefined distance and a far field diffraction pattern with ten-fold rotation symmetry can be observed
